# Spinal canal infection caused by *Streptococcus suis* in human: a case report

**DOI:** 10.1186/s12879-022-07353-y

**Published:** 2022-04-12

**Authors:** Lejia Xu, Xiaoyun Wang, Ziying Lei, Jianyun Zhu, Yihua Pang, Jing Liu

**Affiliations:** 1 Department of Pharmacy, Third Affiliated Hospital of Sun Yat-sen University, Guangzhou, 510630 China; 2 Department of Infectious Diseases, Third Affiliated Hospital of Sun Yat-sen University, Guangzhou, 510630 China; 3 Sun Yat-Sen University, Guangzhou, 510080 China; 4 Key Laboratory of Tropical Disease Control, Sun Yat-Sen University, Ministry of Education, Guangzhou, 510080 Guangdong China

**Keywords:** *Streptococcus suis*, Cerebrospinal fluid, Spinal canal infection, Magnetic resonance imaging, Case report

## Abstract

**Background:**

*Streptococcus suis* is an emerging zoonotic pathogen that mainly causes meningitis, sepsis, arthritis, endocarditis, and endophthalmitis in human. To the best of our knowledge, Spinal canal infection caused by *Streptococcus suis* has rarely been reported.

**Case presentation:**

Here we report a case of spinal canal infection caused by *Streptococcus suis* in a 50-year-old male patient. The patient had a history of close contact with sick pigs days before disease onset. Initially he presented with headache and fever. After admission, the patient began to experience lower back pain, which led physicians to perform a lumber puncture. Meta-genomic next generation sequencing helped identify *Streptococcus suis* in the cerebrospinal fluid. MRI imaging indicated a spinal canal infection caused by *Streptococcus suis*.

**Conclusions:**

Spinal canal infection is an uncommon disease of *Streptococcus suis* infection. This case report indicates that people presented with fever, headache and lower back pain should also be suspected as *Streptococcus suis* infection, especially for those who have had a history of sick pig contact.

## Background

*Streptococcus suis* (*S. suis*) is an emerging zoonotic pathogen that was first isolated from pigs in 1963 [1]. It was later confirmed that this pathogen can be transmitted from pigs to humans mainly through close contact with sick pigs and pork [2]. Before 2005 more than 200 cases were reported worldwide [3]. And according to a meta-analysis that included studies from 1980 to 2015, more than 900 cases were reported [4]. *S. suis* infection is predominantly endemic in pork consuming and pig rearing countries in South-East Asia, imposing a significant health burden in human and causing great financial losses in the swine industry in these countries. A study analyzing the health and economic burden of *S. suis* infection in Thailand in terms of years of life lost estimated that the infection incurred 769 years of life lost (14% of predicted years of life lived if infection had not occurred), 826 quality-adjusted life years (QALYs) lost (21%) and 793 productivity-adjusted life years (PALYs) lost (15%), which equated to an average of 2.5 years of life,

2.6 QALYs and 2.5 PALYs lost per person [5]. *S. suis* ranks the first cause of meningitis in South-East Asia. An epidemiologic study in Bali, Indonesia from 2014 to 2017 found that of 71 acute bacterial meningitis cases during this period, *S. suis* was confirmed in CSF culture of 44 patients [6].

Compared with other common causes of community-acquired bacterial meningitis such as *Streptococcus pneumoniae*, *Haemophilus influenzae* and *Neisseria meningitidis*, the case fatality rate of *S. suis* meningitis is lower [4, 7]. But hearing loss is more common in *S. suis* meningitis. The incidence of hearing loss in *S. suis* meningitis can be as high as 53%, significantly higher than those of pneumococcal meningitis and meningococcal meningitis [4, 8].

*Streptococcus suis* can cause a wide range of clinical manifestations in human. According to a systematic review and meta-analysis conducted in 2014 that included studies through December 2012, the main clinical syndrome was meningitis (pooled rate 68.0%), followed by sepsis, arthritis, endocarditis, and endophthalmitis [9]. Spinal canal involvement in *S. suis* infection has been mentioned in pigs which showed leukocyte infiltrates in histologic examination [10]. But to the best of our knowledge, spinal canal infection caused by *S. suis* has rarely been reported in human.

Here we present the case report of Spinal canal infection due to *S. suis* in a 50-year-old man treated at our hospital’s infectious diseases department. Publication of the clinical data was approved by the patient.

## Case presentation

Here we report a case of a 50-year-old male diagnosed with *S. suis* spinal canal infection. The patient was a pig farm owner with a right-eye vision loss due to injury 30 years ago. He had a history of alcohol use but denied having any chronic disease. He did not have any wounds on his extremities. The patient presented to our department with a 4-day fever and headache, with a temperature of 38.6 °C accompanied with sore throat and dry cough. Apart from these, He did not have other symptoms.

**Fig. 1:**
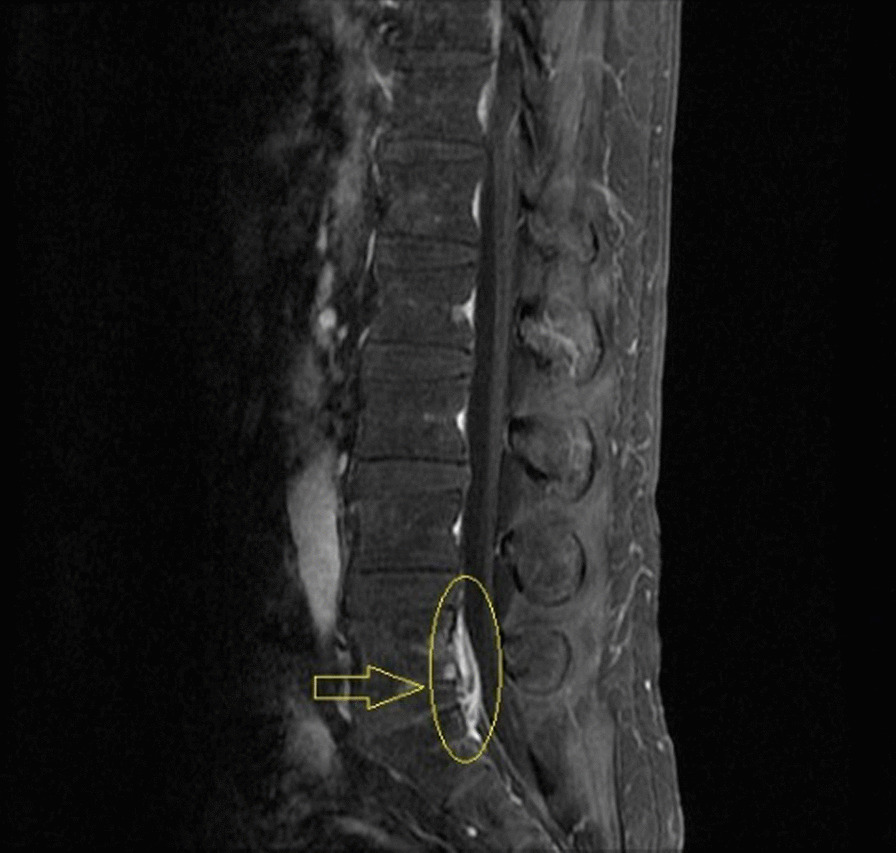
Sagittal T1w image after lower back pain deteriorated showed a large area of high-density signal (as is pointed by the arrow) at L5/S1 level, which reflected spinal canal infection due to *Streptococcus suis*

Right after disease onset he sought for medical assistance in a local hospital where the diagnosis was assumed to be an upper respiratory tract infection. Then oseltamivir and doxycycline were prescribed. After taking these medicines, his symptoms did not improve significantly. To search for further treatment, he came to our hospital. On initial physical examination, in addition to a moderate elevated body temperature and faster heart rates, little moist crackles could also be heard in the left lower lung. The patient also had mild edema in both Lower limbs. Apart from these, other abnormal signs were not observed. Routine blood analysis revealed a thrombocytopenia. WBC count was within normal range. Procalcitonin and C-reactive protein concentrations were 3.08 ng/mL, 83.5 mg/L respectively. Erythrocyte sedimentation rate was 29 mm/H. Chest CT scan showed inflammation infiltrates on the lower left lung, consistent with what was observed on physical examination. The day after admission, the patient complained of Lower back pain, neck pain and a worsening headache. A Lumbar puncture was performed which showed clear CSF. The CSF pressure was 240(mmH_2_O). The WBC count was 40/μL. Biochemical analysis of the CSF revealed that the levels of glucose, chloride and protein were 2.98 mmol/L, 120.2 mmol/L, 1854 mg/L, respectively. Blood culture and CSF culture were both negative. At this time, the patient was treated with ceftriaxone, an empirical treatment for community-acquired bacterial meningitis. Six days after admission, the pathogen was identified through Meta-genomic next generation sequencing, indicating a *S. suis* infection. Then antibiotic was switched from Ceftriaxone to penicillin. After treatment switching, the patient’s symptoms were partly relieved but not completely. He still had a fever. And 5 days after penicillin treatment, the patient experienced a worsening lower back pain. MRI scan shows high density signal in spinal canal of L5-S1 level (as is shown in Fig. 1). A repeated lumbar puncture after symptom worsening revealed lower CSF pressure relative to the previous value. But CSF WBC count rose to 80/μL. Biochemical studies demonstrated that the levels of glucose, chloride, and protein were 3.39 mmol/L, 122.8 mmol/L, and 1126 mg/L, respectively. Considering possible drug resistance to penicillin, the physician decided to switch the treatment to vancomycin according to antibiotic sensitivity data, after which the patient’s symptoms had a greater relief. But 2 days after vancomycin treatment, the patient present with excessive peeling of fingertips, which was probably attributed to vancomycin. Therefore, the antibiotic treatment was switched to Linezolid again. The patient’s symptoms were much more relieved after Linezolid treatment. Auxiliary examinations also indicated a recovery. The patient was discharged after 26 days’ hospitalization.

**Fig. 2:**
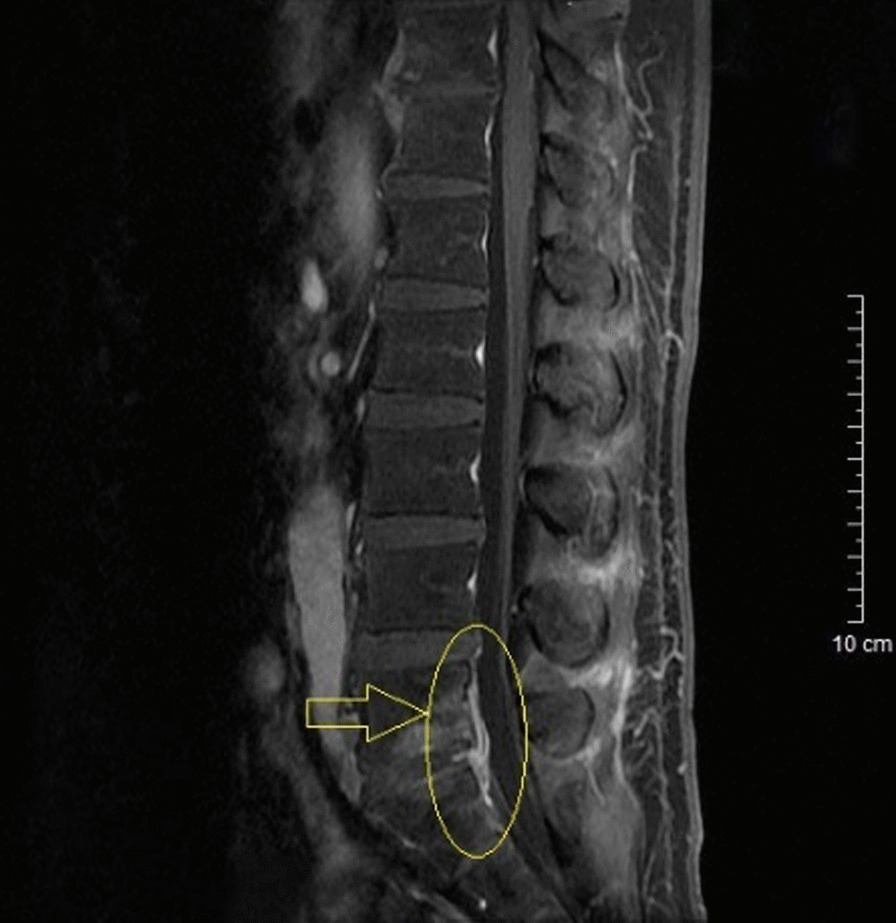
Sagittal T1w image of the patient more than 1 month after discharge demonstrated that high density signal area had contracted (as is pointed by the arrow) and compression to the surrounding tissues had significantly improved compared with what was seen in Fig. 1, consistent with symptom relief

Two weeks after discharge, the patient returned to our hospital for follow up. He did not show any abnormal signs. Hearing loss was not observed either. Whole blood cell count revealed that platelet count had returned to normal. MRI scan after discharge showed improvement of spinal canal infection (as is seen in Fig. 2).

## Discussion and conclusion

*Streptococcus suis*, a zoonotic pathogen that naturally inhabit the upper airways, gastrointestinal tract and genitals of pigs [11], can cause a wide variety of diseases in pigs and humans, including meningitis, septicemia and endocarditis. In human beings, the most common manifestation of *S. suis* infection is meningitis, with sepsis ranking the second common disease [12, 13]. Hearing loss is the most common sequela after recovery from purulent meningitis, whereas death often follows septic shock [3]. Compared with meningitis and sepsis, other clinical presentations, including enteritis, arthritis, endocarditis, pneumonia, spondylodiscitis, endophthalmitis, uveitis and peritonitis, are less common [13]. Initially in this case, the patient presented with typical meningeal signs, such as headache, fever. Signs of spinal canal infection occurred on the 5th day of disease onset, manifesting as Lower back pain, which led the physician to suspect meningitis and perform a lumbar puncture, thus helping find the infectious agent. Epidemiologic history, Metagenomic next-generation sequencing and MRI scan together helped confirm that this is a case of *S. suis* infection that manifested as spinal canal infection. Epidemiology history, such as close contact with sick pigs or pork, is very useful in diagnosing *S. suis* infection [3]. In the outbreaks of *S. suis* infection that occurred in China, almost all the human patients had a history of direct contact with infected pigs or pork [3]. However, there are several cases that develop *S. suis* infection without known history of pig contact [14, 15]. As with many bacterial infections, the confirmation of *S. suis* infection relies on the isolation of the infectious agent from normally sterile body fluid, including blood and cerebrospinal fluid. But the sensitivity of these standard microorganism diagnostic methods is not that satisfying and is affected by antibiotics use prior to sample obtaining, which has been well elucidated in bacterial meningitis [16–18] Traditional serotyping methods such as coagglutination or agglutination tests using serotype-specific antisera are simple, but the production of specific antisera is time-consuming, expensive, laborious, and only available in reference laboratories. Cross-reaction also occurred in some serotypes. So Traditional diagnostic methods are routine, sophisticate, and sometimes insensitive to adequately diagnose the infectious agents [19]. To better identify the causative agents, several molecular biological techniques such as PCR, real-time PCR or WGS have been applied for diagnosis [19–21]. A study designed to verify the usefulness of multiplex PCR in identifying *S. Suis* infection demonstrated that this technique can detect *S. suis* directly from positive hemocultures and CSF, with high sensitivity, specificity compared to culture and serotyping methods. In addition, mNGS, an unbiased approach that is able to detect many potential infectious agents in a single array [22], represents a promising one. A study published in 2019 to verify the real-life usefulness of mNGS in intracranial infection indicates that clinical metagenomic NGS of CSF represents a potential step forward in the diagnosis of meningoencephalitis [22]. In *S. suis* infection, there are also several case reports making use of mNGS to identify *Streptococcus suis* in cerebrospinal fluid and blood samples [21, 23] It is worth noting that in these cases, conventional microorganism detection methods are usually negative. But currently it remains difficult to roll out this technology in countries with limited resources. A previous study developed the immunochromatographic banding (ICS) technique to detect *S. suis* antigens in urine and could be a good option for rapid diagnosis [24]. But results of this study need to be further verified.

It is generally thought that *S. suis* zoonoses result from a wound infection or ingestion of pork contaminated with this pathogen. Raw pork consumption, exposure to pigs or pork, pig-related occupation, male sex are the main risk factors of *S. suis* infection [25]. A case–control study conducted in Vietnam suggests that risk factors of *S. suis* infection include eating “high risk” dishes, including such dishes as undercooked pig blood and pig intestine, occupations related to pigs, and exposures to pigs or pork in the presence of skin injuries [26]. In addition to direct contact with infected pigs or contaminated pork, growing evidence have supported that *S. suis* can also be an airborne pathogen [27–29]. As for the transmission route of *S. suis* in this patient, although the patient had no apparent wounds on his hands or other parts of his body that may predispose him to *S. suis* infection, he did develop such an infection. According to the patient’s initial symptoms such as sore throat and dry coughing, a rational explanation is that the pathogen entered the host through inspiration and invaded the host through respiratory tract mucosa, just as it usually does in pigs, indicating that this zoonotic agent has become much more adaptive to humans.

*Streptococcus suis* is sensitive to many antibiotics, including penicillin, ceftriaxone. But in this case, although mNGS did not provide information about drug resistance, the patient’s response to penicillin treatment indicates that this strain of *S. suis* might not be sensitive to penicillin.

In conclusion, this is a rare case that manifested as spinal canal infection caused by *S. suis*. Although *S. suis* can cause systematic infection, spinal canal infection is an uncommon disease in *S. suis* infection. To the best of our knowledge, there are few reports about this disease caused by *S. suis* in human. We present this rarely-seen case in an aim to expand the clinical spectrum of *S. suis* infection thus help clinicians recognize that patients present with fever, headache and lower back pain may also be a *S. suis* infection, especially for those who have had a history of close contact with sick pigs or pork.

## Data Availability

The data that support the findings of the current study are available from the corresponding author upon reasonable request.

## Abbreviations

CSF: Cerebrospinal fluid
mNGS: Metagenomic next-generation sequencing
*S. suis*: *Streptococcus suis*
MRI: Magnetic resonance imaging
QALYs: Quality-adjusted life years
PALYs: Productivity-adjusted life years
